# Eating and Drinking

**Published:** 1862-05

**Authors:** 


**EATING AND DRINKING.**—There is not, in all the boundless realms of nature, a more beautiful exhibition of the wisdom and goodness of God, than is found in the mechanism of the human body, and the means employed for its growth, its renovation, its preservation, and its well-being. All motion involves friction, and friction necessitates waste and loss. We can not walk a step, nor bend a finger, nor wink the eye, nor think a thought, without there being a less amount of solid matter belonging to the body, as a part of its living substance, than there was before the process commenced; and unless the "wear and tear" is supplied, exhaustion inevitably follows. It is no trouble to understand that, if a man works hard for several hours, he feels weak and tired; and that a good, hearty meal marvelously removes the debility. Only hard students know fully how great is the exhaustion of the entire man, from eight, ten, or a dozen hours of intense thought, without any physical exercise whatever. A good dinner, with an entire "abandon" of thought, renovates and renews both mind and body. This is a clear proof that thought produces waste, as well as physical exertion; it also shows the fallacy, the mischievous fallacy, of a certain class of persons, who have forced themselves on the public as their teachers, that students do not require as substantial food as do common workmen. If great thinkers do not eat and digest substantial beef and bread, they will soon cease to shine as lights in the world of mind. There is not a consistent vegetarian that lives whose name, and work, and memory, for all that is truly good, will not rapidly rot, as certainly as did Jonah's gourd. If a man works or thinks hard for a day, without eating or drinking, he will weigh, at night, from two to five pounds less than he did twenty-four hours earlier. This waste is supplied by food and drink; the most considerable, in bulk and weight, being the latter. A man eats and drinks for two reasons: to supply waste and to keep him warm. Hence the more waste there is, the more we work or think, the more we must eat; the colder it is, also, the more must we eat and drink. In the hottest summer, in the treeless desert, the heat of the body is ninety-eight degrees; on the top of an iceberg, in the Arctic sea, it is no colder, it is no warmer—it is just the same. The temperature of the body then being the same, in health, under all circumstances, the amount of heat to be supplied, the food to be eaten, must be variable; at the same time, it should be proportioned to the amount of heat needed—just as we make a larger or a smaller fire in our dwellings, to meet the temperature without. The man who would order the same amount of fuel to be consumed every day, from November to May, would be considered daft. The man who eats as much to-day as he did yesterday, although it is thirty degrees warmer, is quite as unwise. But there are greater fools than he; and they are those who, as the weather becomes warm, and finding they have not as keen an appetite for food as in the winter, begin to think that something is the matter with them; that they are about to get sick; and to prevent it, begin to "take something," to "whet up the appetite," in the shape of tonics, bitters, whisky, gin, etc. For wise and benign purposes, it was not left to man's wisdom—seeing that few indeed have any in this regard—to daily alter the amount of food and drink taken, according to the variability of the weather and the work done; but instead are appointed various instincts and appetites, self-operating, and beyond our control. In the first place, the appetite declines as the weather becomes warmer; and secondly, it seeks for articles of a different quality. In winter, we love meats and fats, and starches and oily substances, and sweetmeats of every description, because from a half to four fifths of these articles are heat-producing, are fuel for the furnace of life. We not only eat them, but have an appetite for large quantities of them. When summer comes, we not only can not partake of them freely—we crave other things; we want sweets no longer; but are eager for the acid berries and fruits, and tomatoes, and melons. We no longer want heating, solid food, but the cooling and watery vegetable. Sugar and sago, and arrow-root and corn-starch, and fresh meats, have from thirty to forty per cent of solid, heat-producing substances; while fats, suets, oils, and beans and rice, have nearly three times as much. But turning to the vegetables, and salads, and greens, and fruits, and berries, and melons, of spring and summer and autumn, which we so eagerly crave, and in whose consumption we luxuriate, there is a marked difference. Beans have eighty-eight per cent of solid substance, while of the potato seventy-four parts are water, and out of one hundred pounds of berries, over ninety-nine pounds are water—not one single pound of solid heat-producing substance! The mathematical proportions being for peaches nine tenths of one per cent; cherries, six tenths of one per cent; gooseberries and strawberries, about one per cent; and all this is simply because the human body does not require fire to warm it in summer; but water to cool, water to supply the material for perspiration and evaporation.

Such a large part of the foods which we crave in summer being water, these, together with the common beverages of coffee and tea, taken at breakfast and supper, supply as much water for the system as a healthy man requires in ordinary circumstances. Hence a person in good health, and in the moderate pursuit of business, does not feel like drinking water, even in summer-time; is not very thirsty. In fact, great, habitual thirst in summer is the sign of a depraved appetite, resulting from bad habits; or it is a proof of internal fever; and the indulgence of even so simple a thing as drinking cold water largely in summer-time, especially in the early part of the day, will produce a disordered condition of the system. Most persons have experienced more or less discomfort from drinking largely of cold water. If we drink a great deal, we must perspire a great deal; this perspiration induces a greater evaporation of heat from the surface than some have to spare; the result is a chill, then comes the reäction of fever. Many a person arises from the dinner or tea-table, in June, chilly, because too much cold fluids have been taken. From the whole subject, several important, practical lessons are taught:

1. It is unphilosophical to take tonics, bitters or stimulants, to whet up the appetite in warm weather; it is fighting against nature, and can not be done with impunity.

2. In warm weather, live mainly on vegetables, fruits, berries, and coarse breads.

3. Hard work, whether of body or mind, requires substantial and nourishing food.

4. Those who drink little or nothing, even of cold water, in summer, until the afternoon, will be more vigorous, more full of health, and much more free from bodily discomfort, than those who place no restraint on their potations.

